# A Deep Neural Network-Based Multi-Frequency Path Loss Prediction Model from 0.8 GHz to 70 GHz

**DOI:** 10.3390/s21155100

**Published:** 2021-07-28

**Authors:** Chi Nguyen, Adnan Ahmad Cheema

**Affiliations:** SenComm Research Lab, School of Engineering, Jordanstown Campus, Ulster University, Newtownabbey BT37 0QB, UK; nguyen-c@ulster.ac.uk

**Keywords:** path loss, path loss modelling, channel modelling, path loss prediction, AI, DNN, deep neural network, 5G

## Abstract

Large-scale fading models play an important role in estimating radio coverage, optimizing base station deployments and characterizing the radio environment to quantify the performance of wireless networks. In recent times, multi-frequency path loss models are attracting much interest due to their expected support for both sub-6 GHz and higher frequency bands in future wireless networks. Traditionally, linear multi-frequency path loss models like the ABG model have been considered, however such models lack accuracy. The path loss model based on a deep learning approach is an alternative method to traditional linear path loss models to overcome the time-consuming path loss parameters predictions based on the large dataset at new frequencies and new scenarios. In this paper, we proposed a feed-forward deep neural network (DNN) model to predict path loss of 13 different frequencies from 0.8 GHz to 70 GHz simultaneously in an urban and suburban environment in a non-line-of-sight (NLOS) scenario. We investigated a broad range of possible values for hyperparameters to search for the best set of ones to obtain the optimal architecture of the proposed DNN model. The results show that the proposed DNN-based path loss model improved mean square error (MSE) by about 6 dB and achieved higher prediction accuracy R2 compared to the multi-frequency ABG path loss model. The paper applies the XGBoost algorithm to evaluate the importance of the features for the proposed model and the related impact on the path loss prediction. In addition, the effect of hyperparameters, including activation function, number of hidden neurons in each layer, optimization algorithm, regularization factor, batch size, learning rate, and momentum, on the performance of the proposed model in terms of prediction error and prediction accuracy are also investigated.

## 1. Introduction

Determining the radio propagation channel characteristics in different environments is necessary for network planning and the deployment of wireless communication systems [1]. The radio propagation in a physical environment affects the performance of the wireless communication system as the radio waves may experience fading. During propagation, signals suffer from attenuations over distance and frequencies, which are known as large-scale fading and small-scale fading, because of surrounding physical objects as well as atmospheric conditions [2], which make the signals reach the receiver by different paths and experience significant losses. This paper focuses on developing a large-scale fading model which plays an important role in estimating radio coverage, optimize base stations, allocate frequencies properly, and find the most suitable antennas [3]. Large-scale fading due to path loss (PL) and shadowing is caused by distance and the deviation of the received signal due to the presence of obstacles, respectively [4]. The model of large-scale fading is also called as path loss model.

The emerging demands in new wireless technologies and scenarios require new operating frequencies and an increase in data traffic which makes the traditional linear path loss models are not sufficient anymore to capture measured path loss and needs an innovative method for better path loss modelling and prediction. Fundamentally, traditional path loss model approaches are deterministic, stochastic, and empirical models. A deterministic path loss model is a site-specific model, which often requires the information of the environments and often completes a 3-D map propagation, such as the ray tracing model. The deterministic model repeats its calculations when the environment changes, therefore is accurate but high computational complexity. A stochastic model considers environments as a random variable and suffers from a limited accuracy due to some mathematical expression and the probability distribution is added to the model [5]. The empirical model is only based on measurements and observations, such as the Hata model and COST 231 model, which is easy to apply but does not obtain as high accuracy as the deterministic model [3]. The Hata path loss model is applied for frequencies from 150 MHz to 1500 MHz, in which the transmitter heights from 30 m to 200 m and receiver heights from 1 m to 10 m, over 1 km distance were considered. Environments like open, suburban, and urban (both urban medium and urban large) were considered in this model to observe their radio propagation characteristics. The COST-231 is an extension of Hata model with the frequency up to 2000 MHz [6]. Many other empirical path loss models are derived by fitting path loss models as linear log-distance models with the measured data [4]. The problem of traditional path loss models is accomplished by a vast amount of measurement in a particular environment to obtain a certain model; plus long-distance PL models are not fit well with the data in some regions as in [7]. Machine learning (ML) is an alternative approach beyond the traditional large-scale fading models and successfully assists to predict path loss in various operating environments. ML model provides a good generalization on propagation environment because the ML-based models are determined from the data that is measured from the environment [8]. Besides ML, the use of deep learning, including artificial neural network (ANN) and deep neural network (DNN), for path loss model has been considered and proved to obtain better results than traditional path loss [1,3,7,8]. In [9], the ANN-based path loss model produces better performance metrics than ML-based path loss models, including support vector regression and random forest. Deep learning can extract the features from high-dimensional raw data via training by using many layers [1,10]; while in the traditional model, the feature extraction is often not possible, such as COST 231 for the urban environment in terrestrial wireless networks [11], or simplified feature as only the percentage of building occupation [12]. DNN-based path loss model has the advantage that it does not depend on a pre-defined mathematical model, which is different from traditional methods [13]. DNN model has been applied for various environments includes urban, suburban, and rural, or even typical harsh environment as mine [14]. In [15], at each specific frequency path, a DNN model is built for five types of environments urban, dense urban, suburban, dense suburban, and rural areas. Also, the DNN model is built for many ranges of frequency from ultra-high frequency (UHF) network to super-high frequency network [3] as well as for different link types including line-of-sight (LOS) and non-line-of-sight (NLOS) in [16]. 

Path loss based DNN model is divided mainly into two categories in terms of data, mainly into two categories in terms of data, including image data which is mainly derived from satellites [1,3,17] and measured path loss data in real scenarios [7,9]. This paper considers the DNN-based model based on data from channel measurements in real scenarios. 

Almost all of the recent studies have focused on apply neural networks to predict the measured path loss at a single frequency separately or center frequency of various bands [18] and then make a comparison of prediction performance between the traditional path loss model and ML path loss model. DNN shows it can be a more effective model in estimation performance compared with the polynomial regression model and work well with high-dimensional than conventional methods [7]. Longitude, latitude, elevation, altitude, clutter heigh, the distance of unmanned aerial vehicles (UAVs) are the input features to predict the path loss using an ANN at 2100 MHz and 1800 MHz in [9] and in [19] correspondingly. The ANN model is proved to outperform both long-distance model and machine learning methods including support vector machine (SVM) and random forest (RF) in [9]. In [19], ANN architecture adopting a hyperbolic tangent activation function (simply called Tanh activation function) and 48 hidden neurons produced the least prediction error and significantly improve the prediction performance compared to HATA, EIGI, COST-231, and ECC-33 path loss models. In [20], the analogous work is implemented in a smart campus environment at 1800 MHz with two hidden layers neural network and the prediction performance is concluded to outperform the random forest methods. The features to predict path loss using ANN are divided into few groups that include building parameters, information of line-of-sine (LOS) transmission between transmitters and receivers, and cartesian coordinates of both the transmitter and the measurement point, and corresponding distance for NB-IoT operating frequencies (900 MHz and 1800 MHz) [21]. The result shows that ANN provides a higher accuracy compared to the random forest method due to its ability to extract features of the environments. DNN has been proved its superiority in terms of higher prediction accuracy compared to conventional methods and previous machine learning methods by using environment parameters such as terrain clearance angle (TCA), terrain usage, vegetation type, vegetation density near the receiving antenna [22] or atmospheric temperature, relative humidity, and dew point data [18]. DNN-based path loss models are implemented for different frequencies, but each different frequency is fed in the DNN model and observed separately [7,23,24]. Path loss is predicted based on two features distance and frequency at 450 MHz, 1450 MHz, and 2300 MHz in a suburban area [25] and 3.4 GHz, 5.3 GHz, and 6.4 GHz in an urban environment [7]. The result shows that the accuracy of the ANN model is higher and more flexible than that of a close-in path loss model, a two-rays model, and a gaussian process model. In [26], apart from distance and frequency parameters, wall attenuation and floor attenuation are used as input of the ANN model to predict path loss in multi-walls environment GSM, UMTS and Wi-Fi frequency bands. Evaluated model performances show a high improvement in terms of accuracy compared to a calibrated multi-wall model. Clearly, DNN proves its superiority compared to traditional path loss and ML path loss at single frequency separately, however, whether DNN works well with a wide range of frequencies simultaneously and provides better prediction performance compared to a linear multi-frequency path loss models such as ABG path loss model in is [4] still an open issue. Multi-frequency path loss model is the model that measured frequencies will be analyzed and obtain the model at the same time. Motivated by this, this paper will develop a DNN path loss model for a wide range of frequencies (13 frequencies from 0.8–70 GHz) simultaneously and compared the prediction performance of the DNN-based path loss model with ABG path loss model.

Tuning hyperparameters of a deep learning model to find an optimal architecture based on a given dataset is an important process in developing a DNN model. Some studies assume that a neural network is a shallow network with only one hidden layer for the reason that one hidden layer is sufficient for path loss prediction problems [3,13,24,27]. Other studies manually set up the number of the hidden layer at two or three hidden layers; because these studies argue that two or three hidden layers are enough to approximate almost non-linear function between input and output, and a small number of hidden layers can reduce the complexity of DNN model [7,25]. However, there is no rule for the size of the DNN model, including the number of hidden layers and the number of hidden units. Selecting the size of the DNN model is an experimental process. In some cases, some hyperparameters such as activation function, learning rate, and optimizers are fixed in the DNN model for manually tuning. The activation function is fixed as Tanh function as in [24], or one optimization Levenberg-Marquardt algorithm is selected [9]. It is time-consuming and much based on an experiment to manually tune the DNN model [28]. Therefore, some optimization tuning techniques are introduced such as random search and grid search to overcome these problems. In [9], the grid search method is applied to optimize the DNN models, but neither specifies the range of the hyperparameters nor considers learning rate, batch size, epochs, or regularization factor. However, grid search is a time-consuming method, which is only suitable for a few hyperparameters [28]; this paper uses a random search tuning approach and aims to observe with many hyperparameters of the DNN model with a wide range of values of tuning.

Model from signal measurement data rarely mentioned the learning curve which provides information of the behavior of DNN model (loss and accuracy) for on both training set and testing set. This learning curve is important evidence to determine the performance of DNN in terms of accuracy and generalization. In [24,26] a curve of the training set during training is illustrated, but no curve of the testing dataset is described. To overcome this shortage in building a fully connected DNN model, this paper observed the performance of the proposed DNN model in terms of loss and accuracy according to hyperparameters during both training and testing processes.

The contributions of the paper include:

(1) We proposed a feed-forward DNN to model the measured path loss data in a wide range of frequencies (0.8–70 GHz) in urban low rise and suburban scenarios in wide street in case of NLOS link type. By using the random search method, we optimized hyperparameters of the proposed DNN with a broad range of search values. The number of hidden neurons is searched in a range of 100 values and similarly for number of hidden layers in a range of 10 values. The optimized DNN model is proved to be not in case of overfitting or underfitting on training and testing datasets.
(2) The performance of the proposed DNN model for multi-frequency path loss data is compared to the conventional linear ABG multi-frequency path loss model [4] in terms of prediction error and prediction accuracy.
(3) The paper applied the XGBoost technique to analyze the feature importance of the dataset to observe how much each feature contributes to the prediction model.
(4) The effect of each hyperparameter on the performance of the proposed DNN model in terms of prediction accuracy and the prediction error is also investigated.

This paper is organized as follows: in Section 2, we introduce the general principle of the proposed DNN model, including its architecture and hyperparameters. In Section 3, the general information of the multi-frequency path loss model is given and the MMSE method, which is applied to find ABG parameters is described. Section 4 provides information on the use case and data collection. Section 5 focuses on training DNN with the dataset, including pre-processing data, finding feature importance using XGBoost, tuning the model by random search with the training dataset. Finally, the performance metrics of the test set are determined on both DNN model and ABG model for comparison. In Section 6, we present the effect of each parameter on the model is observed independently and Section 7 concludes the paper.

## 2. Proposed Model

A DNN consists of multiple (deep) layers of neurons, which are inspired by the human brain, to conduct a learning task such as classification, regression, clustering, pattern recognition, and natural language processing (NLP) [29]. Multiple layers with multiple neurons make DNNs have the capability to learn a non-linear relationship between input data and output data, especially in the case of a big dataset with multi-dimensional features [7,26]. This is because DNNs can learn high-level features with more complexity and abstraction compared to shallow neural networks [30]. DNNs are categorized into five main types of networks as described in [29], and this paper proposed a feed-forward (FF) multilayer perceptron (MLP) network architecture to estimate the path loss of wireless channels in a wide frequency range from 0.8 GHz to 70 GHz. MLP is a type of feed-forward ANN and plays a role as a base architecture of deep learning (DL) or DNN. In this paper, we consider a fully connected MLP network, a typical MLP network architecture, in which each neuron of a layer connects to each neuron of the next layer [31]. The goal of a feed-forward MLP model is to adjust matrices of weights and bias via training process, which includes two elements a forward feature abstraction and a backward error feedback, to minimize the error between the targets and the predicted values in the output of DNN [32]. Although several studies applied the feed-forward DNN model to estimate path loss, however, DNN models were proposed based on a single frequency path loss dataset only and model performance was evaluated using single frequency path loss models like close-in path loss model [3,7,15]. In this paper, we aim to develop a new DNN model based on multi-frequency dataset which can allow the simultaneous prediction of path loss in multiple operating frequencies or bands. This section provides background on the proposed DNN architecture and approach used to determine the hyperparameters of the proposed DNN model based on dataset.

### 2.1. Architecture

**Figure 1:**
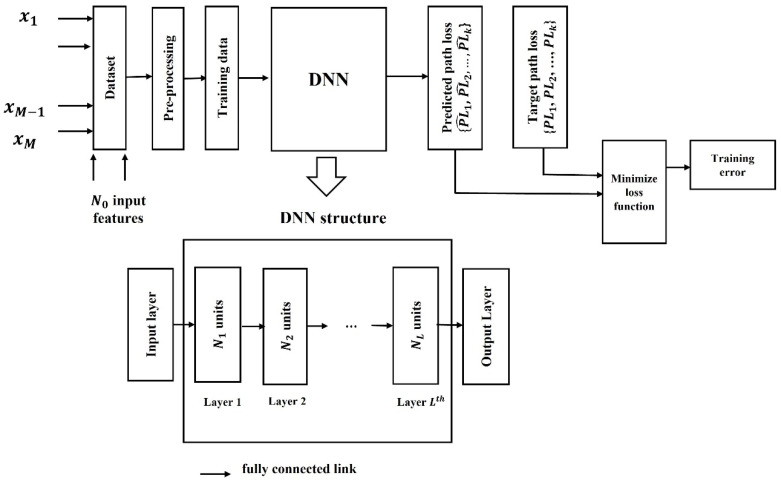
The process of training path loss dataset with the proposed DNN model.

A fully connected MLP DL model is a fully linked network in which all neurons at a certain layer are connected to all neurons of the following layer. This network contains dense layers including one input layer, several hidden layers, and one output layer. The process of training path loss dataset with the proposed DNN model is illustrated in Figure 1.

The input data X = {x_1_, x_2_, …, x_M_} consist of M data samples which are pre-processed and divided it into two groups: (a) training dataset with k samples (or datapoints) and (b) testing dataset with N_0_ input features. The training dataset along with features are fed into the input layer of the DNN network and further processed by L number of hidden layers. Here, each hidden layer contains $N_{l}\;$ units or neurons and each neuron receive the input from neurons of all previous layers then performs a simple computation (e.g., weighted summation) to transform a non-linear relationship between the input and output. To elaborate the operation of a $n^{\mathrm{th}\;}$ neuron at $l^{\mathrm{th}\;}$ layer, (1≤l≤L), output of the neuron, $x_{n}^{\left[l\right]}$, in the DNN network can be written as:

(1) $$x_{n}^{\left[l\right]}=f_{n}^{\left[l\right]}\left(z_{n}^{\left[l\right]}\right),$$

where $f_{n}^{\left[l\right]}$ is the activation function of the $n^{\mathrm{th}\;}\;$ neuron at $l^{\mathrm{th}\;}$ layer and $z_{n}^{\left[l\right]}\;$ is the weighted sum of outputs from all neurons of ${\left(l-1\right)}^{\mathrm{th}\;}$ layer and is expressed as:

(2) $$z_{n}^{\left[l\right]}=\sum_{k=1}^{N_{l-1}}w_{k,n}^{\left[l-1,\;l\right]}x_{k,n}^{\left[l-1\right]}+b_{n}^{\left[l\right]},$$

where $w_{k,n}^{\left[l-1,\;l\right]}$ is the weight connected the $k^{\mathrm{th}}$ neuron at ${\left(l-1\right)}^{\mathrm{th}}$ layer to the $n^{\mathrm{th}\;}$ neuron at $l^{\mathrm{th}\;}$ layer, $b_{n}^{\left[l\right]}$ is bias of the $n^{\mathrm{th}\;}$ neuron at $l^{\mathrm{th}\;}$ layer and $N_{l}$ is total number of neurons at $l^{\mathrm{th}\;}$ layer. The activation function $f_{n}^{\left[l\right]}$ can be one of the following commonly used activation functions such as logistic sigmoid, Relu, and Tanh and are expressed in mathematical form as follows:

(3) $$f_{s}\left(x\right)=\frac{1}{1+{\;e}^{-x}}\;\;,$$

(4) $$f_{r}\left(x\right)=\max\left\{0,x\right\},$$

(5) $$f_{t}\left(x\right)=\frac{e^{x}-e^{-x}}{e^{x}+e^{-x}}\;\;,$$

where $f_{s}\left(x\right),{\;f}_{r}\left(x\right)\;$ and $f_{t}\left(x\right)$ are the sigmoid, Relu, and Tanh functions on an input parameter x, respectively. 

The predicted path loss at the output of the DNN model is expressed by a k-elements vector ${\hat{y}}^{\left[O\right]\;}$=$\;{\hat{\{\mathrm{PL}}}_{1}$, ${\hat{\mathrm{PL}}}_{2},\;\ldots ,{\hat{\mathrm{PL}}}_{k}$} and compare with the targeted values of path loss {${\mathrm{PL}}_{1}$, ${\mathrm{PL}}_{2}$, …, ${\mathrm{PL}}_{k}$} for model training. The predicted path loss value from each neuron at the output of the output layer is defined as:

(6) $${\hat{y}}^{\left[O\right]}=f^{\left[O\right]}\left(z^{\left[O\right]}\right),$$

where $f^{\left[O\right]}$ is the activation function of $n^{\mathrm{th}\;}$ neuron at the output layer and $z^{\left[O\right]}\;$ is the weighted summation of outputs from all neurons in the last hidden layer $L^{\mathrm{th}\;}$ and is defined as:

(7) $$z^{\left[O\right]}=\sum_{k=1}^{N_{L}}w_{k,n}^{\left[L,O\right]}x_{k,n}^{\left[L,O\right]}+b_{n}^{\left[O\right]},$$

where $w_{k,n}^{\left[\;L,\;O\right]}$ is the weight that connects the $k^{\mathrm{th}}$ neuron of $L^{\mathrm{th}}$ layer to the $n^{\mathrm{th}\;}$ neuron of the output layer, and $b_{n}^{\left[O\right]}$ is bias of $n^{\mathrm{th}\;}$ neuron at the output layer. The difference between predicted path loss and the target path loss (also known as label) is defined by loss function J. The goal of deep learning is to minimize the loss function by using optimization algorithms to find the parameters, particularly weights and biases, for the DNN model. The loss function J is a function of parameter θ=(W,b) in which W is the weight matrix of the DNN model and b is the bias matrix of the DNN model. The adjustment of weights and biases of the DNN is conducted in the backpropagation step. There are several approaches to update the parameters of the DNN model which depends on the optimization algorithms. For example, considering gradient descent, the basic optimization algorithm of DNN, the repeatedly updating weights, and bias are based on taking the derivatives of J(θ) with respect to every weight and biases of the DNN model [7] as defined by Equations (8) and (9):

(8) $$z^{\left[O\right]}=\sum_{k=1}^{N_{L}}w_{k,n}^{\left[L,O\right]}x_{k,n}^{\left[L,O\right]}+b_{n}^{\left[O\right]},$$

(9) $$b^{\left[l\right]}\leftarrow b^{\left[l\right]}-\alpha \frac{\partial J\left(\theta \right)}{\partial b^{\left[l\right]}},$$

In this paper, the model parameter θ=(W,b) only considers updating weights W to reduce the computational complexity for the model. In addition, $w_{k,n}^{\left[l\right]}$ is a weight in a neural network between $k^{\mathrm{th}}$ and $n^{\mathrm{th}}$ neurons at layer $l^{\mathrm{th}}\;$ in a weight matrix of the DNN model.

### 2.2. Hyperparameters

To find the parameters of the DNN model, including weights and biases, the hyperparameters of the DNN models must be set up before implementing the training process. Although hyperparameters are parameters that we cannot determine during the training process, they contribute to establishing the structure of the model through the number of hidden layers which decide the DNN model size and the number of neurons (units) of each layer. In addition, hyperparameters also decide the efficiency and accuracy of model training via parameters such as learning rate (LR) of the gradient descent algorithm, activation function, regularization factors, or types of optimization algorithms [28]. Hyperparameters tuning and fining optimal configurations is challenging and time-consuming in a deep learning model [33]. Most widely used methods for hyperparameter selection are based on experience in training deep learning models; however, it is lack of logical reasoning and difficult to find an optimal set of hyperparameters for a DNN model with a given dataset. To overcome the experience tuning method, auto-machine learning (AML) is proposed as a technique to train and design a deep learning model with a trade-off of additional computation requirements. Hyperparameter optimization (HPO) is a key technique in AML which is useful in searching for optimized hyperparameters for a deep learning model. In the literature, several techniques have been used to tune hyperparameters for the DNN mode such as grid search and random search. Grid search is a simple and parallelism method in which all possible combinations of hyperparameters values are tuned; therefore, it is time-consuming and only applicable for a few hyperparameters [28]. Meanwhile, random search is a parallelism HPO that saves much time compared to grid search by randomly searching the combination of hyperparameters. Particularly, a random search can combine with the early stopping technique, an approach that can avoid overfitting problems in the backpropagation model [34]. Although random search can overlook some combinations and may not promise optimum parameters however with enough searching iterations it can solve the variance problem and can provide a better selection of optimized parameters for the optimized model [28]. The search space in random search is bounded for hyperparameters while in case of grid search the search space is over all possible grid points. Random search randomly sample points in search domain, and grid search evaluate every sample points in the grid [35]. In this paper, random search is selected as an HPO method to find the structure of the proposed DNN model because we will be tuning hyperparameters with a large number of combinations. The hyperparameters of the proposed feed-forward fully connected DNN model are composed of the number of hidden layers, the number of units in DNN models, the learning rates, the regulation parameter λ, the optimization algorithms used to update the weight matrix and activation functions. In this paper, the number of units in each hidden layer is observed in two different cases: (1) when the number of hidden units are same in all hidden layers, and (2) different hidden layers can have different number of hidden units.

## 3. ABG Path Loss Model and Parameters

Multi-frequency path loss model ABG, ${\mathrm{PL}}^{\mathrm{ABG}}\left(f,d\right)$, in dB is expressed as [4]:

(10) $${\mathrm{PL}}^{\mathrm{ABG}}\left(f,d\right)\left[\mathrm{dB}\right]=10\alpha \log10\left(d/d_{0}\right)+\;\beta +\log10(f/1\;\mathrm{GHz})\;\mathrm{for}\;d\geq d_{0}$$

where $d_{0}=1\;m$ which is suggested as a physical-based reference distance and d is the distance between the transmitter and the receiver. The parameters α and γ present the dependence of path loss on a distance between the transmitter and receiver and a measured frequency, respectively. The parameter β is an optimized offset (floating) value for path loss in dB. The empirical path loss model is defined as:

(11) $$\mathrm{PL}\left(f,d\right)\left[\mathrm{dB}\right]={\;\mathrm{PL}}^{\mathrm{ABG}}\left(f,d\right)\left[\mathrm{dB}\right]+{\;X}_{\sigma }^{\mathrm{ABG}},\;\mathrm{for}\;d\geq d_{0}$$

where $X_{\sigma }^{\mathrm{ABG}}$ is the shadowing factor, which describes large-scale fluctuations around the mean path loss over distance d and has a distribution of a Gaussian random variable N~(0,σ) with zero mean and standard deviation σ [4].

The difference between the empirical path loss and the ABG path loss model can be defined as given in Equation (12). The error e is minimized to estimate the ABG path loss model parameters as given in Equation (13) [4]:

(12) $$e=\mathrm{PL}\left(f,d\right)-{\;\mathrm{PL}}^{\mathrm{ABG}}\left(f,d\right),$$

(13) $$\left(\begin{array}{c} \alpha \\ \beta \\ \gamma \end{array}\right)={\left(\begin{array}{ccc} \overset{N}{\underset{i=1}{\sum }}D_{i}D_{i} & \overset{N}{\underset{i=1}{\sum }}D & \overset{N}{\underset{i=1}{\sum }}D_{i}F_{i}\\ \overset{N}{\underset{i=1}{\sum }}D_{i} & N & \overset{N}{\underset{i=1}{\sum }}F_{i}\\ \overset{N}{\underset{i=1}{\sum }}D_{i}F_{i} & \overset{N}{\underset{i=1}{\sum }}F_{i} & \overset{N}{\underset{i=1}{\sum }}F_{i}F_{i} \end{array}\right)}^{-1}\left(\begin{array}{c} \overset{N}{\underset{i=1}{\sum }}D_{i}B_{i}\\ \overset{N}{\underset{i=1}{\sum }}B_{i}\\ \overset{N}{\underset{i=1}{\sum }}F_{i}B_{i} \end{array}\right),$$

where $B={\mathrm{PL}}^{\mathrm{ABG}}\left(f,d\right)\left[\mathrm{dB}\right]$, $D=10\log_{10}\left(d\right),$ and $F=10\log_{10}\left(f\right)$. The number of total measured data points is equal to N.

The multi-frequency path loss model ABG will be used to compare with the proposed DNN path loss model performance based on performance matrices (more details can be found in Section 5.3) as both models are simultaneously strong candidates for multiple frequencies path loss.

## 4. Use Case and Dataset

The dataset is considered as presented in [36]. It has been measured in three countries (UK, South Korea, and Japan) from 0.8 GHz to 73 GHz in different environments including urban-low rise, urban high-rise, and suburban environments. The urban high rise is an environment in which signals travel through high buildings of several floors each, while the urban low rise is the one with wide streets and low building heights e.g., residential area. Regarding the heights of stations, which are transmitter and receiver, two NLOS scenarios including above rooftop and below rooftop are considered in this paper only.

**Figure 2:**
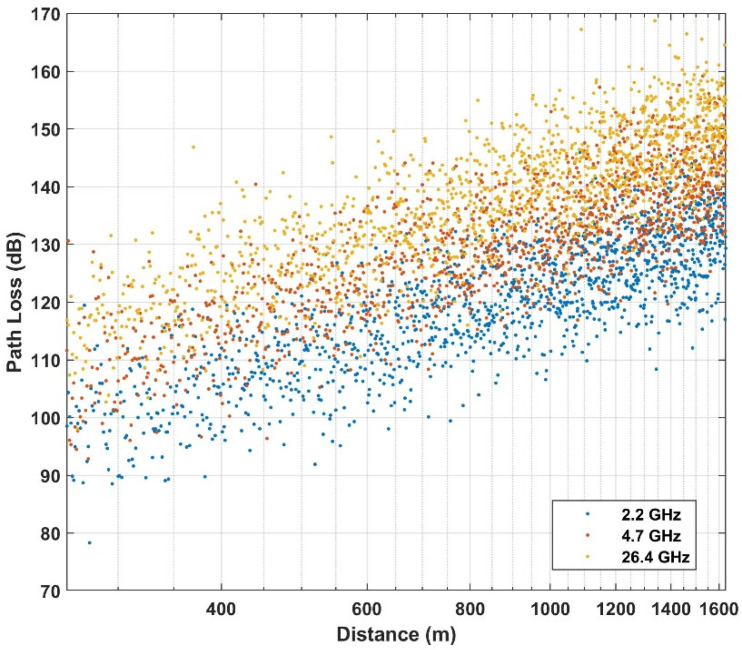
Empirical path loss at an above rooftop in an urban high-rise environment.

**Figure 3:**
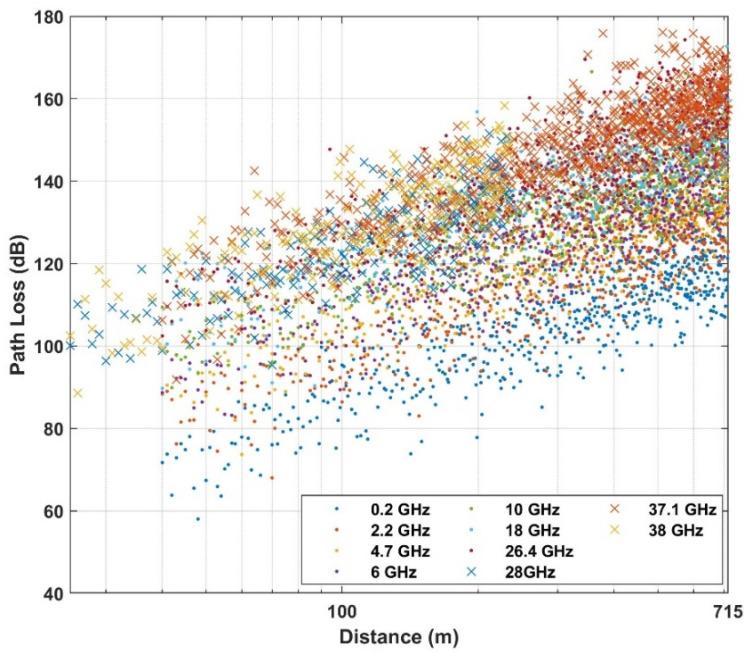
Empirical path loss at below rooftop in urban high-rise environment.

**Figure 4:**
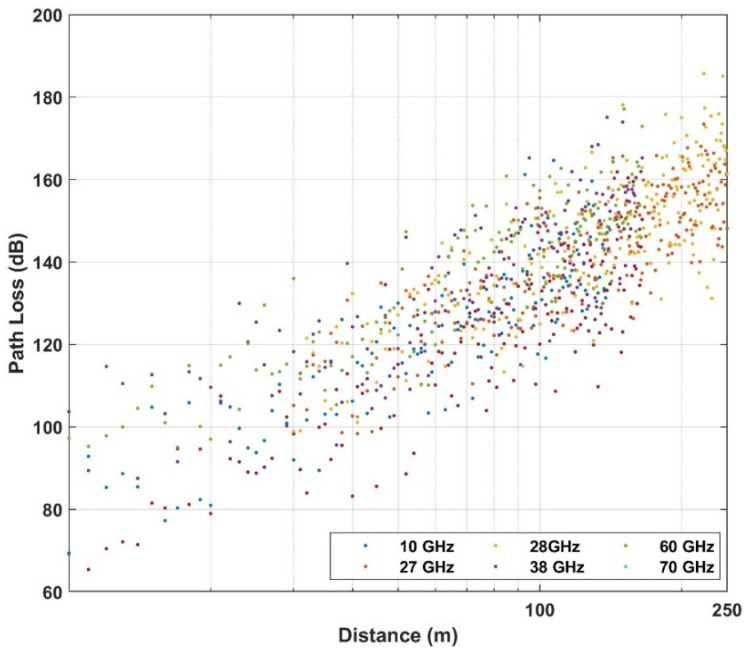
Empirical path loss at below rooftop in an urban low-rise (suburban) environment.

Below the rooftop is a scenario where both transmitter and receiver are under the height of the surrounding rooftop; meanwhile above the rooftop is a scenario in which one base station (transmitter or receiver) is above the surrounding rooftop and the other is under the surrounding rooftop. In this paper, the path loss dataset is considered in the case of NLOS, in both above rooftop and rooftop scenarios and in both urban and suburban environments. The number of measured frequencies is K = 13 which are presented by elements of a vector of frequencies in GHz  with frequencies includes values in f={0.8; 2.2; 4; 6; 10; 18; 26.4; 27; 28; 37.1; 38; 60; 70}. The data set is summarized in Table 1 and shown in Figure 2, Figure 3 and Figure 4 based on different environments.

## 5. DNN Model Training and Validation

### 5.1. Dataset Preparation

The dataset contains measured path loss values obtained from the NLOS link type scenarios as discussed in Section 4 and given in Table 1. The distance range for each frequency depends on the measurement scenarios. The total number of samples or data points are 10,984 in which the input dataset contains four features, including propagation category (below rooftop and above rooftop), environment (urban and suburban), distance, and frequencies while the target output is the values of respectively measured path loss. 

**Figure 5:**
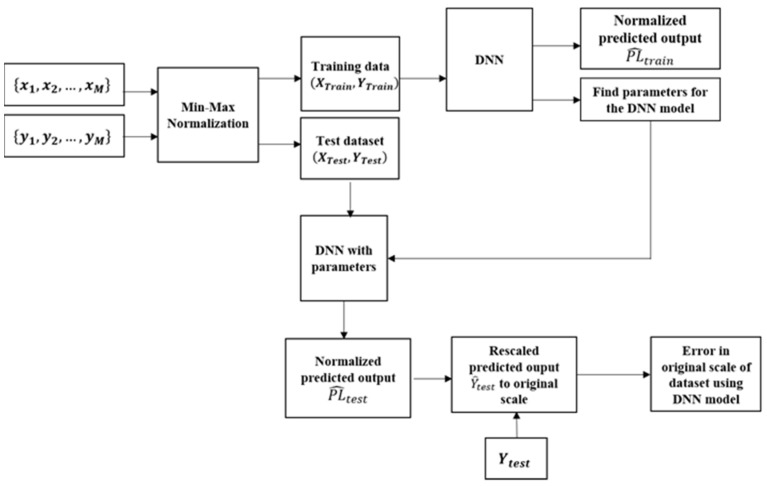
Block diagram of the processing data using proposed DNN model.

The dataset needs to be pre-processed before training by the DNN model as each input sample contains different features e.g., distance and frequency data have a range of different scales, therefore differences in scales can lower the performance of prediction at the output of the DNN model. The performance of the model can be improved using normalization. The process of processing data through the DNN model is demonstrated in Figure 5. Firstly, the whole dataset is randomly split into two groups of training (80%) and testing (20%). Here, the testing set is put separately to evaluate the generalized performance of the DNN model, and test set should be seen one time after the training process.

Normalization is an important process to boost the prediction accuracy at the output of the DNN model. In this paper, four features of the input data are normalized by min-max normalization over the range from 0 to 1 [9]. The normalization is applied for the input of training data set as follows [24]:

(14) $$\;\bar{x}=\frac{x_{\;}-\;\min\left\{x_{\mathrm{train}}\right\}}{\max\left\{x_{\mathrm{train}}\right\}-\;\min\left\{x_{\mathrm{train}}\right\}}$$

where x is an input of training data or testing prior to normalization, $\;\bar{x}$ are the normalization data, and the $x_{\mathrm{train}}\;$ is the input of the DNN model for the training purpose. The label or output values of the training dataset or test set are expressed as:

(15) $$\;\bar{y}=\frac{y\;-\;\min\left\{y_{\mathrm{train}}\right\}}{\max\left\{y_{\mathrm{train}}\right\}-\;\min\left\{y_{\mathrm{train}}\right\}}$$

where y is a label of training data or testing data before normalization, $\;\bar{y}$ are the normalization of the label in the training dataset or the target testing set, and the $y_{\mathrm{train}}\;$ is the target of the training dataset. It is noted that the test set is a dataset that is used one time and is not used to train the model, therefore all the parameters for the normalization process are taken from the training set. 

In Figure 5, the training dataset is used to find the parameters of the DNN model based on minimizing the loss function. The test set applies the proposed DNN model to evaluate the prediction ability of the model on a new dataset or the generalization of the proposed model. The difference between the predicted path loss and the empirical path loss on the test set is evaluated by different performance metrics, more detail is provided in Section 6.3. Since the dataset are normalized with the range value from 0 to 1,the prediction values at the output DNN are also in the range from 0 to 1. Also, the performance of the DNN model regarding loss and accuracy is the range from 0 to 1 as well, and we can consider as normalized performance metrics. To compare with the conventional multi-frequency linear ABG path loss model, predicted path loss values on the test set will be rescaled to the original scale in  dB as ABG path loss model does not use a normalization dataset.

### 5.2. Analysis of Feature Importance on the Prediction Using XGBoost Algorithm

**Figure 6:**
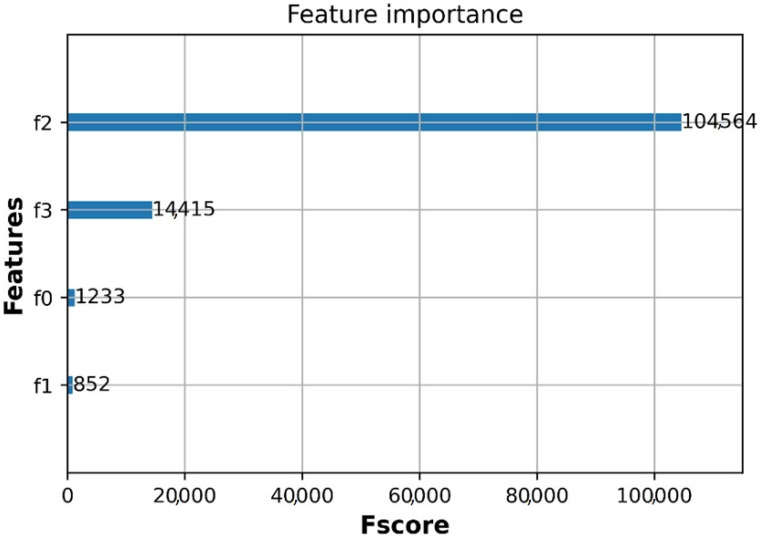
Feature importance using XGBoost algorithm.

In this paper, an extreme gradient descent algorithm XGBoost is applied to analyze the importance level of each feature in the dataset [37]. An algorithm based on a decision tree model, such as XGboost, is able to easily produce the feature importance of the dataset [38]. The feature importance score shows that how useful each feature contributes is to the XGBoost model i.e., highest score defines the highest feature importance [39]. In this article, the data set contains four features f0, f1, f2,  and f3 including propagation category (below rooftop and above rooftop), environment (urban and suburban), distance and frequencies, respectively. As shown in Figure 6, the level of feature importance is represented via the score in which environment (urban or suburban) has the least score.

The most important feature is distance as having the highest score, and the second important feature is frequency. The third important feature is the propagation category (below rooftop and above rooftop) with the minimum score. Distance and frequency are two most important factors that contribute to the path loss prediction, following by propagation category and propagation environment (urban/suburban).

### 5.3. Performance Metrics

Performance matrices are used to evaluate the accuracy of the path loss model including mean absolute error (MAE), mean square error (MSE), root mean square error (RMSE), R2, mean absolute percentage error (MAPE), and maximum prediction error (MaxPE) as given below:

(16) $$\;\mathrm{MSE}=\frac{1}{Q}\overset{Q}{\underset{i=1}{\sum }}{\left({\mathrm{PL}}_{i}-{\hat{\mathrm{PL}}}_{i}\right)}^{2},$$

(17) $$\mathrm{RMSE}=\sqrt{\frac{1}{Q}\sum_{i=1}^{Q}{\left({\mathrm{PL}}_{i}-{\hat{\mathrm{PL}}}_{i}\right)}^{2}},$$

(18) $$\;R2=\frac{\left|{\hat{\mathrm{PL}}}_{i}-\bar{{\mathrm{PL}}_{i}}\right|}{{\mathrm{PL}}_{i}-\bar{{\mathrm{PL}}_{i}}},$$

(19) $$\;\mathrm{MaxPE}\;=\;\max\left|{\mathrm{PL}}_{i}-{\hat{\mathrm{PL}}}_{i}\right|,$$

(20) $$\mathrm{MAPE}=\frac{1}{N}\sum_{i=1}^{Q}\left|\frac{{\mathrm{PL}}_{i}-{\hat{\mathrm{PL}}}_{i}}{{\mathrm{PL}}_{i}}\right|100\%,$$

where ${\mathrm{PL}}_{i}\;$ is the empirical path loss, ${\hat{\mathrm{PL}}}_{i}$ is the predicted path loss at the samples point $i^{\mathrm{th}}$, $\bar{{\mathrm{PL}}_{i}}$ is the mean of the empirical path loss, and Q is the total number of samples that are used to calculate performance metrics.

### 5.4. Training of Proposed DNN Model

The purpose of training the proposed DNN model is to optimize the hyperparameters with given training datasets, from which we can achieve the optimized DNN models. The optimal hyperparameters are obtained during the training process using random search technique by fitting observed models with training dataset to find which is the optimal model. The optimal DNN model will produce a minimum normalized MSE loss and the highest R2 value. The range of different hyperparameters of the proposed model is presented in Table 2, in which hyperparameters such as activation function, number of hidden layers and number of hidden neurons in each layer, optimized algorithms which are applied to update weights and biases, and its learning rate as well as momentum, and L2 regularization factor.

**Figure 7:**
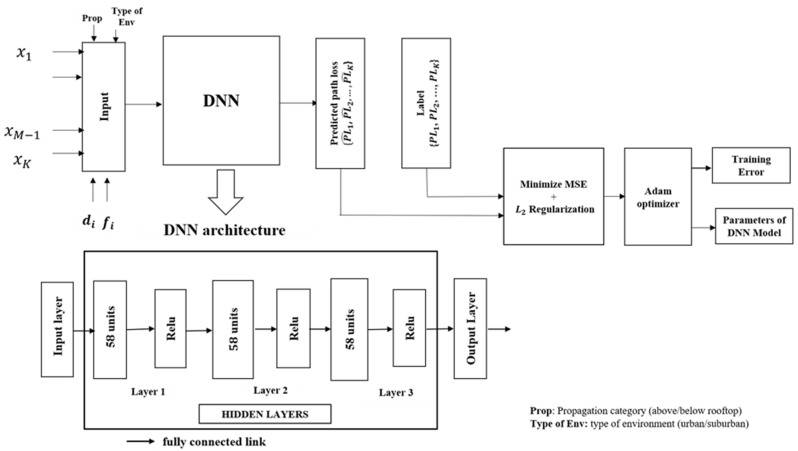
Proposed fully connected DNN model.

To increase the possibility of finding the optimal network size, a large number of hyperparameters are considered to tune DNN model size. This is because there is still no theorem or an approach to determine the size of a DNN model, as it depends on many factors, an important one of which is the size of the dataset. In this paper, the wide range of numbers of hidden layers and number of hidden units are tuned by random search, in which the number of hidden layers ranges from 1 to 10 with a step of 1 layer and the number of hidden units ranges from 1 to 100 with a step of 1 unit. In addition, the DNN size was tuned in two different cases. In the first case, the number of neurons in each layer is kept constant while in the second case the number of neurons in each layer can be different. For the second case, we consider the DNN model with two hidden layers and three hidden layers. In each case, the number of neurons in each layer will be selected from a permutation of the set {10, 20, 30, 40}. Choosing the learning rate will affect testing error of the DNN model. A large value of the learning rate can produce a high convergence error due to high training speed while a small value of the learning rate could slow down the training process [37,38]. In this paper, the learning rate is tuned in two schemes which include constant and adaptive. The constant scheme will keep the same value of the learning rate at 0.0001. In contrast, adaptive learning rate scheme initializes learning rate value at 0.001 and in case there is no change of training loss value (normalized MSE loss) or validation score (R2 score) in two consecutive epochs by threshold error equal to $10^{-5}$, the learning rate will be reduced 5 times. The summary of hyperparameters is provided in Table 2. The optimized DNN architecture is illustrated in Figure 7 after finding the optimal hyperparameters of the model as listed in Table 2.

After applying the random search technique on the given training dataset, the optimum hyperparameters of the DNN model were found, as given in Table 3, where the activation function is the Relu function, the  L2 regularization factor is equal to 0.0001, the number of hidden layers is 3 with 58 units at each layer. The optimization algorithm Adam is used to updating the weight and bias with momentum is equal to 0.4. Adam is an optimizer with an adaptive learning rate algorithm, which is suitable with the path loss dataset of 10,984 points.

**Figure 8:**
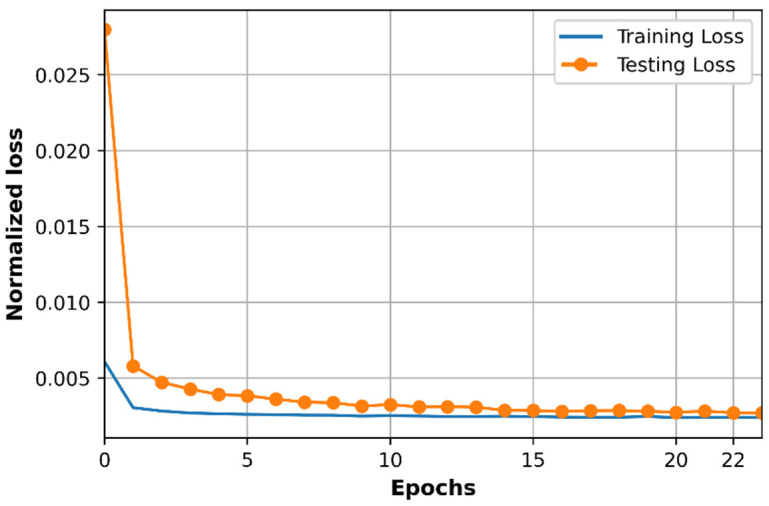
Loss of training and testing datasets according to epochs.

**Figure 9:**
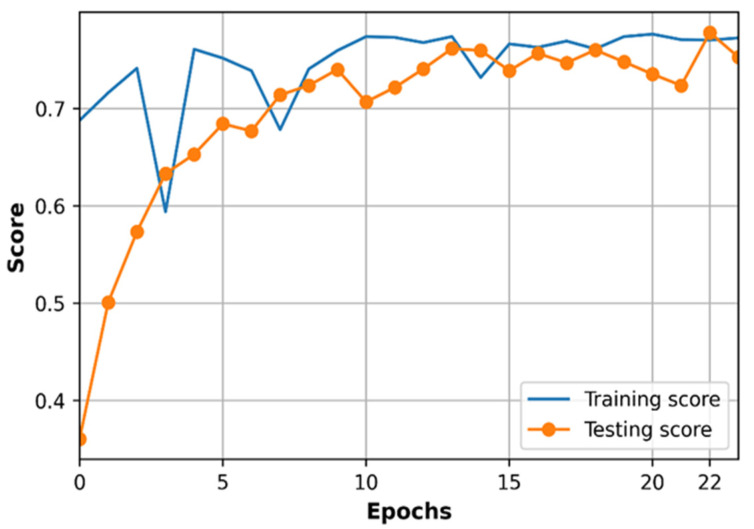
Accuracy of training and testing datasets according to epochs.

The learning curve based on normalized loss (MSE) and accuracy (R2 score) of the DNN model are observed with the training dataset and testing dataset as shown in Figure 8 and Figure 9.

As we can see in Figure 8, the training loss is lower than the testing loss, and both training loss and testing loss are changing at very closed values during training progress. The loss in Figure 8 is the normalized loss which is determined in the range from 0 to 1 since the training dataset and testing dataset are normalized before processing by DNN. The training loss and testing loss curves converge at $22^{\mathrm{th}}$ epochs at the value of MSE loss equal to 0.0023. Path loss prediction is a regression problem therefore we use R2 score to evaluate the performance accuracy of the model as shown in Figure 9. In contrast to loss curves, the accuracy of training data is almost higher than the accuracy of testing data, and the two accuracy curves are at very closed values at $22^{\mathrm{th}}$ epochs, in which R2 is around 0.77 for training and testing datasets, respectively. The curves of loss and accuracy based on training and testing datasets in Figure 8 and Figure 9 shows that the optimized DNN model works well on both the training dataset and testing dataset, which does not cause overfitting or underfitting problems. Underfitting can be observed when the error cannot be minimized during the training phase while overfitting occurs when the error between training and testing dataset cannot be minimized [40]. We can see that the error in the training dataset is small, and the gap between error on the training dataset and testing dataset is small at the convergence point ($22^{\mathrm{th}}$ epoch).

### 5.5. Testing DNN Model

In this section, the prediction accuracy of the DNN path loss model is compared with the linear ABG path loss model. The prediction accuracy is evaluated only on the test set to compare the ability to predict new data (or generalization performance) of the proposed DNN model in comparison to the ABG path loss model. The test set includes 2197 samples that are tested with the proposed DNN model one time and are fed to the ABG path loss model to find the ABG path loss parameters, ABG mean path loss and standard deviation. Applying the ABG path loss model on the test set, the path loss parameters are estimated as α= 3.62, β = 16.45, γ=2.7, and the shadowing of the ABG path loss model follows a normal gaussian distribution with zero mean and standard deviation equal to 8.63 dB. Two models are compared based on the performance metrics as given in Table 4.

The proposed fully connected feed-forward DNN provides about a 6 dB decrease in MSE and an increase of 2% in the accuracy of the R2 score compared to the ABG path loss model. This R2 score is acceptable because in the multi-frequency path loss model the improvement of the R2 score means the average improvement of the R2 score for a total number of measured frequencies simultaneously, particularly in this case this R2 score is for 13 frequencies.

**Figure 10:**
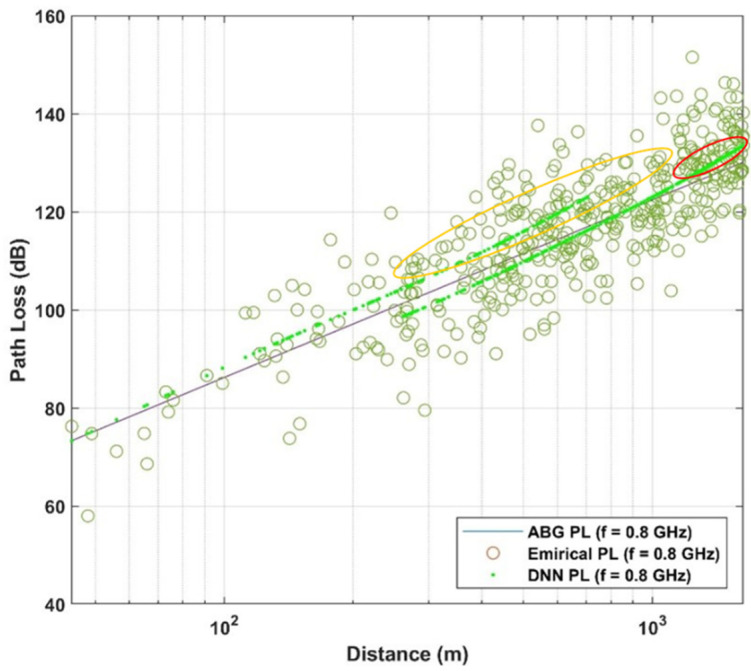
Path loss models and empirical data for low 5G band.

The ABG path loss model and DNN path loss model are further compared in three cases including low 5G frequency band (under 1 GHz), mid 5G frequency band (1 GHz to 6 GHz), and high 5G frequency band (6–100 GHz) [41]. In Figure 10, low 5G frequency band with f=0.8 GHz, DNN model shows its ability to follow the distribution of the data (as highlighted in orange in red circles) while ABG path loss model cannot capture the distribution of the data around the mean ABG path loss.

**Figure 11:**
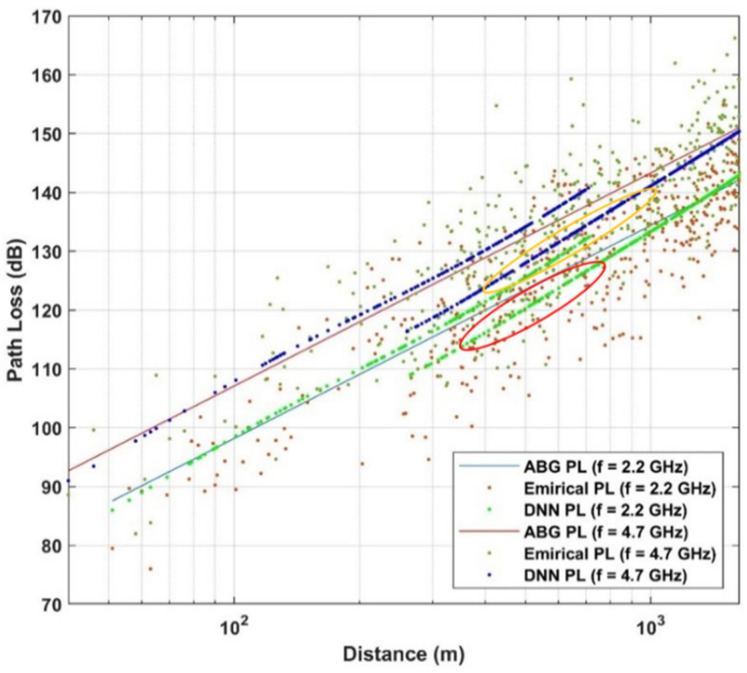
Path loss models and empirical data for mid 5G band.

In the case of the mid 5G frequency band, we have two representative frequencies that are f=2.2 GHz and f=4.7 GHz. Like the case of the low-frequency band, the ABG path loss model keeps a linear fit while DNN always tends to follow the changes in the data distribution. Particularly at f=4.7 GHz, we can observe as highlighted by orange circle in Figure 11, DNN predictions follow the trend in data compare to ABG.

**Figure 12:**
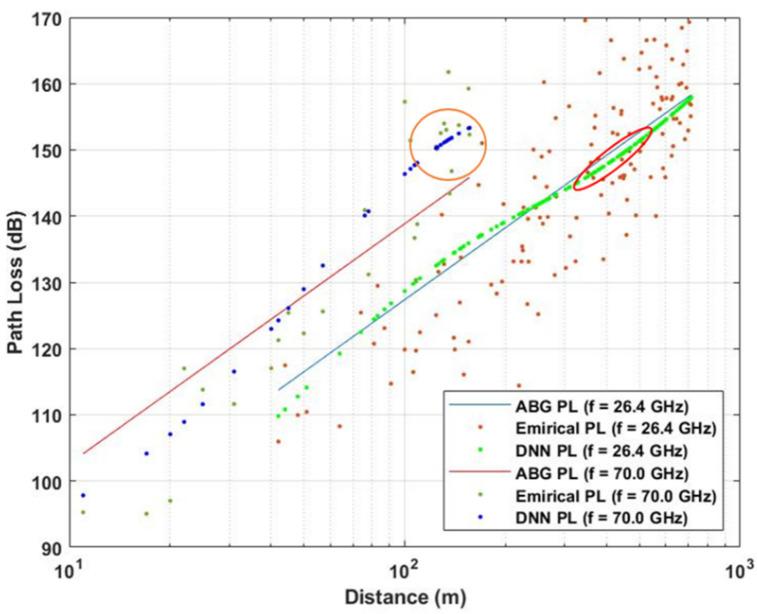
Path loss models and empirical data for high 5G band.

In the case of the high 5G band, even with a smaller number of datasets compared to the two above cases, DNN shows its superiority over the ABG model when there is a change in the distribution of the dataset. We can see at the orange circle in Figure 12, when the data points start to go far from the ABG mean path loss (orange circle), DNN still follows the data trend and present the mean values of these samples.

DNN shows its similar behavior as at low 5G band and mid 5G band and f=70 GHz, in which the curve of DNN will slightly move up and down following the trend of the data distribution at a specific area, that shows the superiority of DNN model to ABG path loss model in term of estimating empirical path loss.

With a trend of a large number of datasets and a greater number of frequencies, the statistical or distribution of datasets will be random and complicated. In this case, DNN can produce a lower estimation error compared to the ABG model because DNN adapts better to the change of the data distribution compared to the ABG model. In summary, DNN path loss is found to be superior to the ABG path loss model in terms of following the change in data distribution and provides a better fitting.

## 6. Impact of Hyperparameters on the Proposed Model Performance

This section analyses how the prediction performance in terms of normalized MSE loss and R2 scores have changed according to change in each hyperparameter, respectively. To do that, we will keep other optimal hyperparameters of the optimized model as given in Table 3 while adjusting one hyperparameter at one observation. The observations will implement for some important hyperparameters such as learning rate, optimizers, activation function, regularization factor, and the number of hidden layers. The optimal model in the case of tuning hyperparameter separately will be decided by the loss using loss curve and R2 score using accuracy curve. An optimal model will produce a minimum loss and probably give the best R2 score. Based on the loss and accuracy values of each hyperparameter tuning case, this section will evaluate the prediction performance of the two methods, the manual tuning in this section, and the random search as in Section 5.4.

### 6.1. Effect of Learning Rate

The learning rate is observed with Adam optimizer and considered to have adaptive values that mean the learning rate will not be constant during the training process. Several values of adaptive learning rate in an adaptive learning rate schedule will be observed. To make it simple to compare, the learning rate initialization is set up to 0.001 and will be reduced 5 times. The learning rate in the adaptive schedule will be in the set of $\left\{0.001,\;0.0002,\;4\times 10^{-5},8\times 10^{-6},\;1.6\times 10^{-6},\;3.2\times 10^{-7}\right\}.$

**Figure 13:**
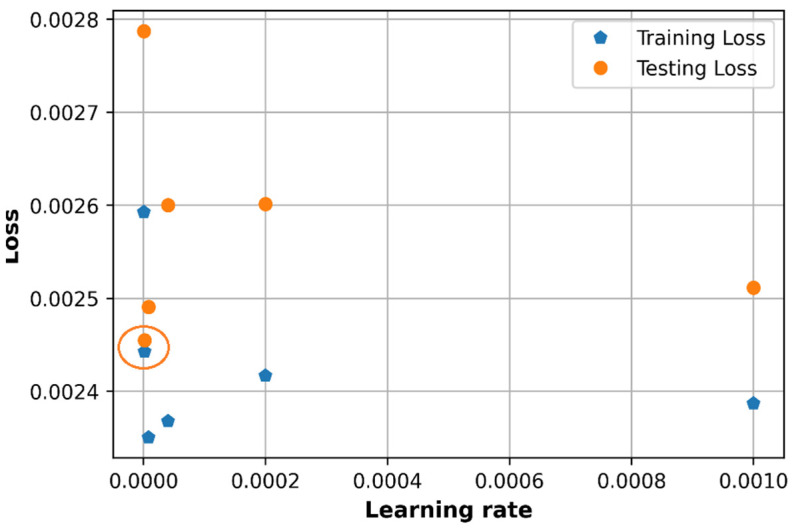
Comparison of loss with different learning rate.

**Figure 14:**
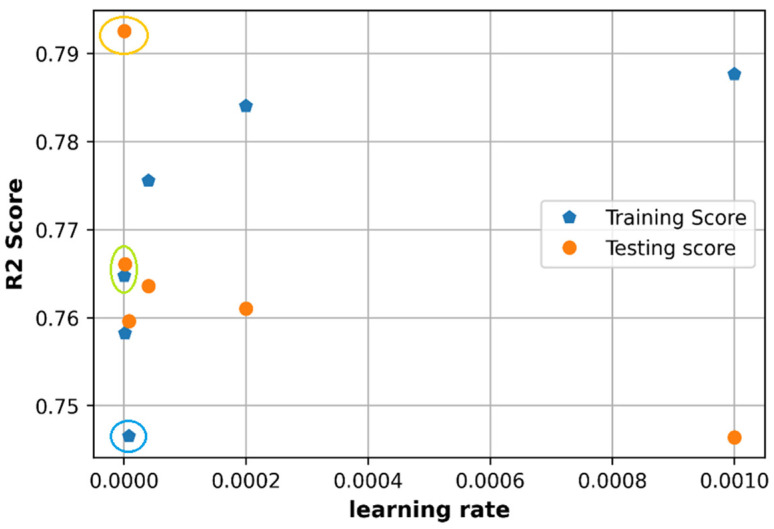
Comparison of accuracy with different learning rate.

As shown in Figure 13 and Figure 14, the learning rate at 0.001 does not produce the lowest loss and in fact provides very low accuracy in the test set. When the learning rate is adjusted from the initialized value by dividing by 5, we can see that at a very small learning rate value that closed to 0, the model can get a relatively low loss as marked in an orange circle in Figure 13 and high accuracy as in orange and green circles in Figure 14. The adaptive schedule learning rate divided by 5 can be applied to get a low prediction error and high R2 score.

The initialized learning rate at 0.001 is a good candidate of initialization value since it does not produce a good performance at first and needs to be adjusted to get a lower error and higher accuracy. The best value of loss in Figure 13 (about 0.00245) is still higher than that in the random search method (0.0023). The best accuracy on the test set in Figure 14 (0.79) is higher than that in random search (0.77), however, at this point (in orange circle) the values of the R2 testing score is much different from the value of R2 training score (in blue circle). The best candidate of R2 score in Figure 14 can be the point at the green circle, which gives an R2 testing score around 0.765 and this value is closed to that in a random search.

### 6.2. Effect of Optimizers

**Figure 15:**
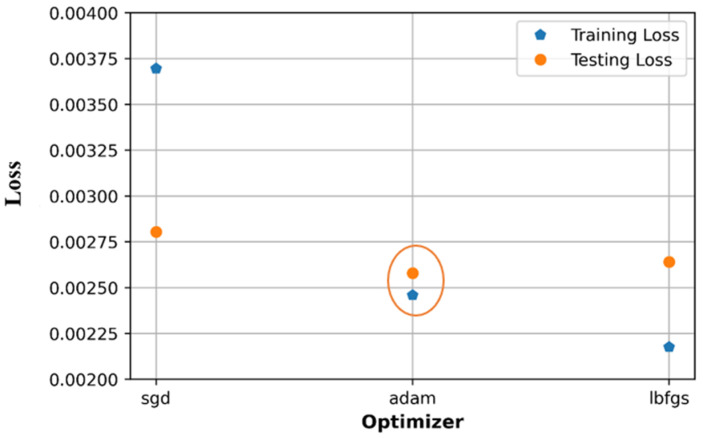
Comparison of loss with different optimizers.

**Figure 16:**
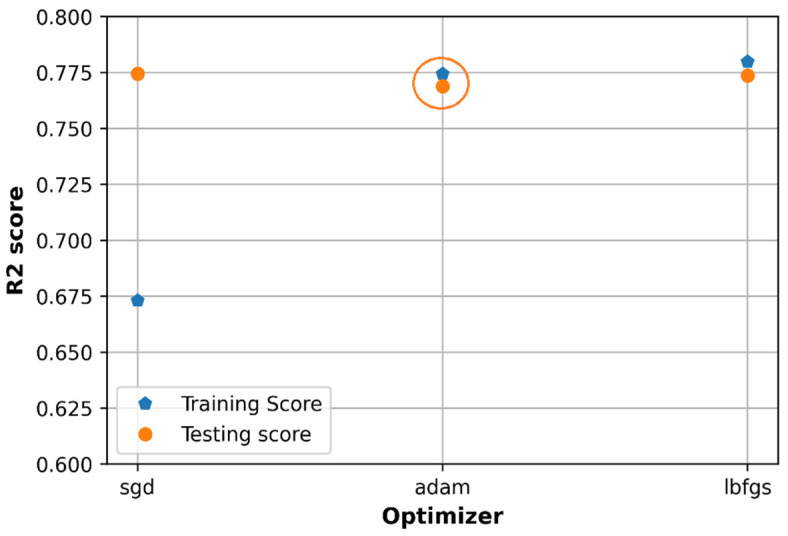
Comparison of accuracy with different optimizers.

There are three optimizers observed to tune the hyperparameters which are stochastic gradient descent (SGD), Adam, and L-BFGS algorithm. As shown in Figure 15, the training loss and testing loss of Adam optimizer get the lowest values compared to those of SGD and L-BFGS; in addition, the training loss value and testing loss value of Adam optimizer are not too different. The training and testing accuracy of the Adam and L-BFGS optimizers are at very closed values as in Figure 16 based on the R2 score. However, Adam is a better option since it shows better performance on MSE loss as shown in Figure 15 (highlighted by orange circle).

The loss on the testing set of both the three optimization algorithms (with a minimum value equal to 0.0026) is higher than that in the random search method (0.0023). The accuracy on the test set has a quite similar value to that in the random search method (about 0.77).

### 6.3. Effect of Activation Functions

**Figure 17:**
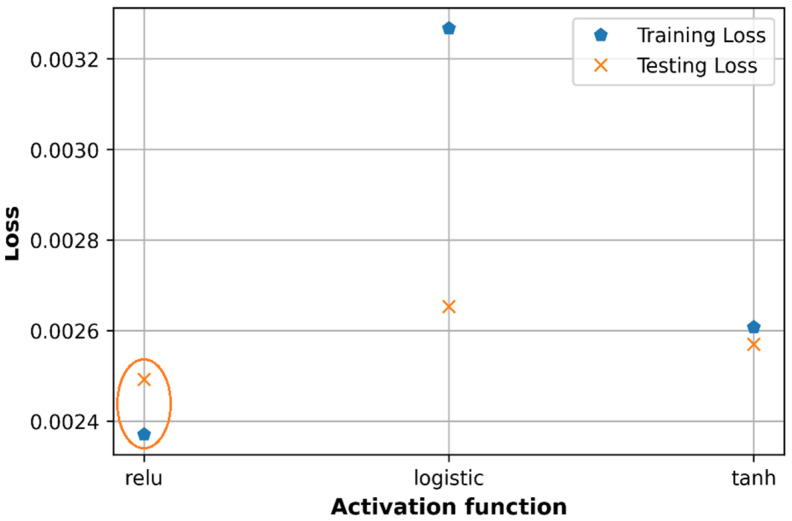
Comparison of loss with different activation functions.

**Figure 18:**
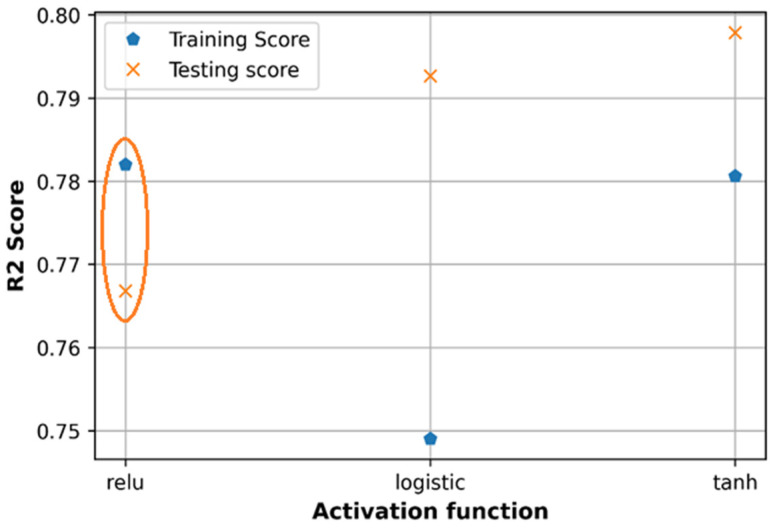
Comparison of accuracy with different activation functions.

The three most popular activation functions are selected for tuning hyperparameters model which includes Relu, sigmoid logistic, and Tanh activation functions. All three activation functions are used to evaluate the performance of the model independently as in Figure 17 and Figure 18. As shown in Figure 17, the difference between training loss and testing loss in the case of Tanh activation function is smaller than that in the case of Relu activation function. In contrast, the training loss is much greater than the testing loss in the case of sigmoid logistic function. The Relu activation function is thus a good candidate because it produces the lowest loss.

With the Relu function, the testing loss is higher than the training loss, while with Tanh function, the testing loss is slightly lower than the training loss. In this paper, the path loss dataset is normalized in the range of [0,1] which seems to match with the output response range of Relu function which is in the range of [0,1] rather than with that of Tanh function which ranges in [−1,1]. Therefore, in both cases of tuning separately or using random search, Relu function is a good option to get low prediction error and high accuracy. Sigmoid logistic activation function gives a higher loss in the training dataset than in the testing dataset, which can cause an overfitting problem, so is not applied in this case.

Regarding the R2 score in Figure 18, Tanh activation function produces a higher accuracy compared to the Relu function on both training and testing datasets however in the case of Tanh function, the accuracy of testing is higher than the accuracy of the training dataset. Regarding the R2 score, the Relu function is a good choice for the proposed DNN model.

### 6.4. Effect of Regularization L2 Factor

**Figure 19:**
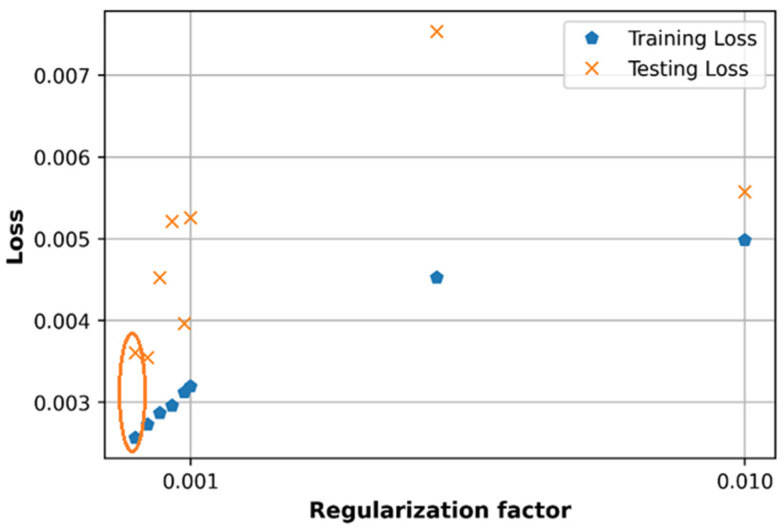
Comparison of loss with different regularization factor.

**Figure 20:**
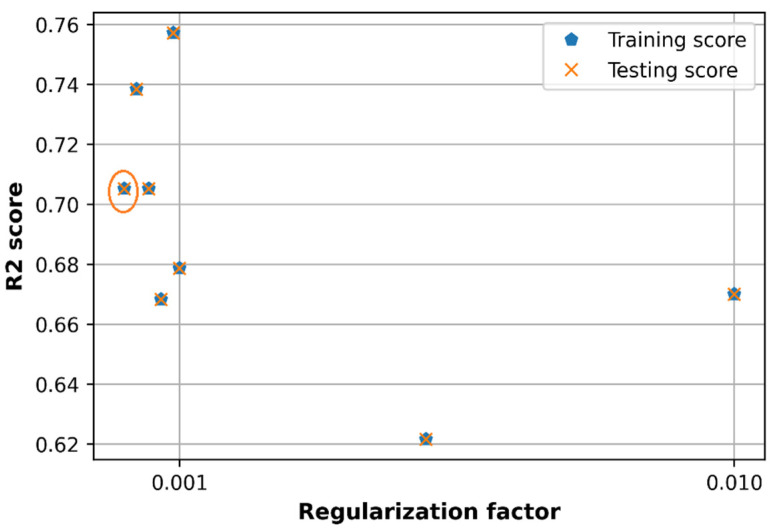
Comparison of accuracy with different regularization factor.

The loss and accuracy are observed versus values of regularization factor in the set [0.0001, 0.0003, 0.0005, 0.0007, 0.0009, 0.001, 0.01] as shown in Figure 19 and Figure 20. The loss in case of λ=0.0001 produces the lowest training loss and testing loss compared to other cases of regularization L2 factor, which can be seen in the orange circles in Figure 19.

In the random search method, the L2 regularization factor is also equal to 0.0001 as in Table 3 and this value can be a good option for the proposed DNN model in the case of the multi-frequency path loss dataset. However, tuning the regularization factor separately produces a R2 score accuracy of 0.71 which is much lower than when using the random search method (0.77).

### 6.5. Effect of Hidden Size

**Figure 21:**
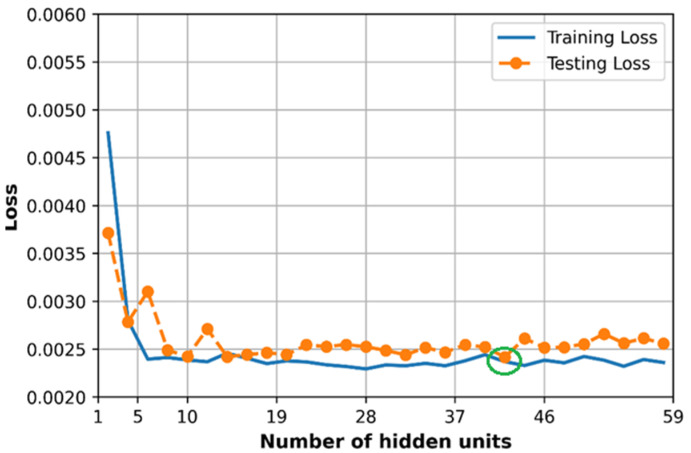
Comparison of loss with different the number of hidden units.

**Figure 22:**
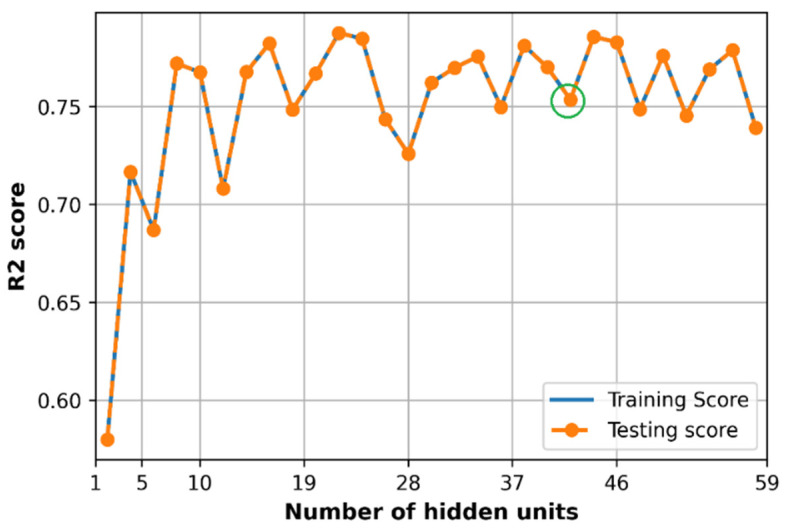
Comparison of accuracy with different the number of hidden units.

The number of hidden layers is considered to be three layers, and the number of hidden units (same at each hidden layer) is observed to evaluate the loss and accuracy of the training and testing dataset. As we can see in Figure 21, the training loss and testing loss converge at a minimum value of 0.0024 at hidden units equal to 41 units which are presented in the green circle, and at this point, the R2 score gets about 0.75 as in the green circle in Figure 22.

The optimal number of neurons in each hidden layer when tuning separately (41 neurons) is different from the random search method result (58 neurons each layer). However, the size of the DNN model in the random search method (in Table 3) produces a slightly lower loss (0.0023) and higher R2 score (0.77) compared to the model size tuned manually in this subsection. 

In summary, tuning hyperparameters individually is useful to see the effects of each parameter on the performance error and performance accuracy of the proposed DNN model. However, tuning hyperparameters using random search produces slightly smaller prediction error and higher prediction accuracy on testing dataset compared to individually tuning of each hyperparameter. Tuning hyperparameters using random search shows its superior to separately tuning method because we can consider many hyperparameters at the same time as well as ensure a good performance in terms of prediction error and prediction accuracy of the optimized DNN model.

## 7. Conclusions

The paper proposed a DNN model to predict path loss based on the measurement data below the roof of both urban and suburban environments in a wide range of frequencies (0.8 GHz to 70 GHz) in the case of NLOS links simultaneously. The proposed DNN model demonstrated that it is superior in predicting the mean path loss compared to the linear ABG path loss model. Random search approach was applied to tune a wide range of hyperparameters (for example 100 values of hidden neurons are tuned) to determine the optimal proposed DNN architectures rather than just a few hyperparameters with a small tuning range. The learning curve in the training process showed that the model is not overfitting or underfitting which shows that the proposed model not only works well on the training dataset but also gives an improved prediction accuracy compared to the ABG path loss model. The paper also considered how each hyperparameter affects the performance of the proposed DNN model in terms of both prediction error and prediction accuracy. 

## Figures and Tables

**Table 1:** Data Sets for NLOS scenarios.

Propagation Category	Environment	Frequency (GHz)	Distance (m)
Below rooftop	Urban high-rise	0.8, 2.2, 4.7, 6, 10, 18, 26.4, 37.1	40–715
		28, 38	25–235
	Urban low-rise (suburban)	10, 60	10–165
		27	10–140
		28, 38	30–250
		70	10–170
Above rooftop	Urban high-rise	2.2, 4.7, 26.4	260–1630

**Table 2:** Tuning values of hyperparameters.

Hyperparameters	Values
Activation function	Sigmoid logistic, Relu, Tanh
Number of units in each hidden layer (same units/neurons in each hidden layer)	Number of hidden layers = [1:1:10] Number of hidden units = [1:1:100]
Number of units in each hidden layer (different units/neurons in each hidden layer)	Two hidden layers, number of neurons in each of two hidden layers are permutations in the set {10, 20, 30, 40} Three hidden layers, number of neurons in each of three hidden layers are permutations in the set {10,20, 30, 40}
Optimized algorithms	Stochastic gradient descent, Adam, RMSprop
L2 regularization factor	0.0001, 0.005, 0.001, 0.01
Batch size	10, 20, 32, 64
Momentum (in case using Adam)	0.0, 0.2, 0.4, 0.6, 0.8, 0.9
Learning rate	Constant (0.0001) Adaptive (initialized learning rate = 0.001)

**Table 3:** Optimized DNN model for path loss prediction.

Hyperparameters	Values
Activation function	Relu
$L_{2}$ regularization factor	0.0001
Batch size	20
Momentum	0.4
Hidden layer sizes and units	(58,58,58)
Learning rate	Adaptive
Optimizer	Adam

**Table 4:** Performance metrics of DNN and ABG path loss model.

Performance Metrics [dB]	DNN	ABG
Max error	39.07	39.92
MSE	68.48	74.56
RMSE	8.27	8.63
MAE	6.45	6.71
R2 score	0.77	0.75
